# Impact of Oral Health Status on the Quality of Life of Patients With Chronic Kidney Disease: An Observational Study

**DOI:** 10.1155/ijod/8881601

**Published:** 2026-03-10

**Authors:** Sérgio Henrique Gonçalves de Carvalho, Jackeline Mayara Inácio Magalhães, Gustavo Gomes Agripino, Dmitry José de Santana Sarmento, Marina Gallottini, Gustavo Pina Godoy

**Affiliations:** ^1^ Department of Dentistry, Paraíba State University, Araruna, Paraíba, Brazil; ^2^ Department of Clinical and Preventive Dentistry, Federal University of Pernambuco, Recife, Pernambuco, Brazil, ufpe.br; ^3^ Department of Dentistry, University of Sao Paulo, Sao Paulo, Brazil, usp.br; ^4^ Department of Pathology, Federal University of Pernambuco, Recife, Pernambuco, Brazil, ufpe.br

**Keywords:** oral health, quality of life, renal failure

## Abstract

**Objective:**

To evaluate the impact of oral health on the quality of life of patients with chronic kidney disease (CKD) before kidney transplantation.

**Methods:**

This is an observational, cross‐sectional, analytical, and descriptive study involving patients with CKD prior to kidney transplantation. Patients underwent oral health assessment and responded to the Oral Health Impact Profile (OHIP‐14), in addition to other data being collected.

**Results:**

Most patients had a high rate of decayed and missing teeth, with a mean DMFT of 15.33 ± 9.37. Periodontal alterations (altered CPI) were present in 42.5% of the sample. Regarding awareness, 68.8% were uninformed about the need for dental care before transplantation. The overall OHRQoL was generally positive (median OHIP‐14 = 1.00; IQR = 5.00). However, significant differences were found between OHIP‐14 domains (*p*  < 0.001), with impacts concentrated in psychological disability and physical pain. Worse OHRQoL scores were significantly associated with lower educational level (*p*  = 0.020) and being on renal replacement therapy (*p*  = 0.030).

**Conclusion:**

Patients awaiting kidney transplantation present a high burden of oral disease but maintain a paradoxically positive perception of their oral health‐related quality of life. This perception is primarily impaired by physical pain and psychological factors, such as embarrassment. The findings highlight the need for early and systematic inclusion of dental care in the multidisciplinary management of CKD, prioritizing patients with lower education and those on dialysis to mitigate clinical risks and improve psychosocial well‐being.

## 1. Introduction

Chronic kidney disease (CKD) is a progressive condition characterized by structural and functional alterations, including decreased kidney function, estimated glomerular filtration rate reduction, and markers of renal damage such as albuminuria, hematuria, or imaging abnormalities persisting for more than 3 months. In Brazil, systemic arterial hypertension and diabetes mellitus (DM) are the main causes of CKD [1–5]. This disease represents a major public health challenge due to its association with increased morbidity, reduced quality of life, and mortality [2, 3, 5]. In advanced stages, renal replacement therapy (RRT) is the therapeutic gold standard, aiming to remove toxins, restore electrolyte balance, and maintain adequate fluid homeostasis, thereby improving prognosis and survival [1, 6, 7].

CKD also produces important repercussions on oral health. Patients frequently present a higher number of decayed teeth and periodontal alterations, such as bleeding on probing, attachment loss, periodontal pockets, xerostomia, mucosal pallor, and uremic stomatitis. Biochemical changes involving calcium, phosphorus, urea, sodium, and potassium levels in saliva may further contribute to oral impairment. Additionally, the prioritization of systemic illness often leads to neglect of oral care, which may negatively impact quality of life [2, 4, 8–10].

Oral health may influence the quality of life of patients undergoing RRT. Thus, dental evaluation is recommended as part of multidisciplinary management to reduce infections, systemic complications, and malnutrition, with consequent benefits to emotional, social, and physical functioning [11]. Oral health‐related quality of life is a multidimensional construct that incorporates functional, psychological, and physical domains, and its assessment contributes to oral health planning. Among the available instruments, the OHIP‐14 is widely used, validated for Portuguese, and applicable in fields such as oncology, nephrology, and stomatology [12]. Its domains—functional limitation, physical pain, psychological discomfort, physical disability, psychological incapacity, social disability, and social disadvantage—enable a broad assessment of oral health influence on daily life, with higher scores indicating worse quality of life [11, 13, 14].

Despite this relevance, there is a paucity of studies evaluating, in an integrated manner, the sociodemographic characteristics, oral health conditions, and their impact on the quality of life of patients with end‐stage renal disease (ESRD), particularly those awaiting kidney transplantation. Consequently, the true clinical significance of oral health parameters during RRT remains unclear, limiting the development of strategies to minimize adverse health outcomes in this population.

Therefore, the objective of this study was to evaluate the impact of oral health on the quality of life of patients with CKD before kidney transplantation. Specifically, it seeks to identify the demographic and epidemiological profile of these patients, assess their self‐perception and oral health conditions, and analyze the association between sociodemographic and clinical variables and the results of the OHIP‐14 questionnaire in order to understand the factors that may influence the relationship between oral health and quality of life in this population.

The hypothesis of the study is that patients with poor oral health conditions and unfavorable sociodemographic characteristics would present worse oral health‐related quality of life, as measured by the OHIP‐14.

## 2. Materials and Methods

### 2.1. Ethical Aspects

This research proposal was forwarded to the Human Research Ethics Committee of the University of São Paulo and UNIFESP (Kidney and Hypertension Hospital) and subsequently approved under Opinion Number 1,824,857 and Opinion Number 2,362,239, respectively, with all the requirements of Resolution 466/2012 of the National Health Council, Ministry of Health, being met. Data collection was started only after approval of the project by the institutional Ethics Committees and after the study participants had voluntarily signed the free and informed consent form.

### 2.2. Study Design

This was an observational, cross‐sectional, inferential, or inductive analytical study of patients with CKD immediately prior to kidney transplantation that used a statistical‐descriptive approach. It should be noted that no artificial intelligence software was used to prepare the manuscript.

### 2.3. Place of Study

The study was carried out at the Institute of Health and Social Action (Campina Grande – PB) and the Kidney and Hypertension Institute (São Paulo – SP) from March 2021 to March 2022.

### 2.4. Population and Sample

The study population consisted of patients with CKD prior to kidney transplantation, who were treated at the aforementioned services during the period of data collection. A nonprobabilistic convenience sample was used, including all eligible patients with CKD awaiting kidney transplantation at the study sites during the data collection period. The sample size was determined by patient availability and guided by similar studies in the literature. No formal sample size calculation was performed due to the exploratory and descriptive nature of the study.

### 2.5. Eligibility Criteria

Criteria for inclusion in the study were (1) presence of chronic end‐stage disease before kidney transplantation, (2) agreement to participate in the study by signing the free informed consent form, (3) age older than 18 years, and (4) physical and mental conditions to participate in the clinical evaluation. Excluded were (1) patients who had already received an organ transplant, (2) patients unable to undergo oral physical examination, and (3) illiterate patients.

### 2.6. Instrument for Data Collection

The main instrument for data collection was the OHIP‐14, which assesses oral health‐related quality of life in the following domains: functional limitation, physical pain/discomfort, psychological impact, and behavioral impact. For collection of the other data, a clinical chart was developed that comprised socioeconomic‐demographic and epidemiological data, harmful habits, medical information, dental history, and oral health assessment by detailed physical examination using forms of the Decayed‐Missing‐Filled Teeth (DMFT) index, highlighting the total score, and the Community Periodontal Index (CPI).

### 2.7. Data Collection Procedure

First, patients treated at the services were approached and received an explanation of the terms of the study and a subsequent invitation to participate. Patients who agreed were invited to sign the free informed consent form. The chart was then filled out, starting with general epidemiological data. Next, the DMFT and CPI forms were applied, recording all data.

Finally, the short version of the OHIP‐14, validated for the Portuguese language, was applied. This questionnaire measures the impact of oral health on the quality of life of affected patients. The participants answered 14 questions by assigning scores to each question that range from 0 to 4, where 0 = never, 1 = rarely, 2 = sometimes, 3 = repeatedly, and 4 = always. The result is obtained as the sum of scores attributed to each question and interpreted as a global score, with higher sum values corresponding to worse quality of life in the OHIP‐14, as the responses to each question were classified ordinally, ranging from best answer (0 = never) to worst answer (4 = always) [12].

All biosafety criteria were strictly followed, and confidentiality of information and anonymity of the subjects were maintained in all data collections.

All variables of interest were assessed using standardized and validated instruments. Sociodemographic and clinical data were obtained from patient interviews and medical records, while oral health status was evaluated through direct clinical examination applying the DMFT and CPI indices, following WHO diagnostic criteria for dental caries and periodontal status, respectively. The primary outcome was oral health‐related quality of life, assessed through the OHIP‐14 questionnaire validated for Brazilian Portuguese. All instruments and measurement procedures were applied uniformly to both exposed and nonexposed groups, ensuring comparability of data collection and interpretation across groups.

### 2.8. Calibration

Calibration was first performed using a study based on scientific articles, in which the correct application of the oral examination and data collection was discussed with the researchers. All parameters evaluated are in accordance with the principles established by the SB2000 guidelines [15]. To assess intraexaminer agreement, the kappa test was used to evaluate physical examination, DMFT index, and CPI at two time points at an interval of 15 days. A kappa value of 0.930 was obtained.

### 2.9. Variables

The main outcome of this study was oral health‐related quality of life, measured by the total score obtained from the Oral Health Impact Profile (OHIP‐14) questionnaire. Higher scores indicated a greater negative impact of oral conditions on quality of life.

The main exposure and predictor variables included oral health conditions assessed through clinical examination using the DMFT index and the Community Periodontal Index (CPI). Additional independent variables included sociodemographic and clinical characteristics such as age, sex, educational level, family income, presence of comorbidities, stage of CKD, time on dialysis, and harmful habits (e.g., smoking and alcohol consumption).

Potential confounding factors were considered to be age, sex, and socioeconomic level, given their possible influence on both oral health status and perceived quality of life. The presence of systemic diseases, such as DM and hypertension, was treated as a possible effect modifier, as these conditions may alter the relationship between oral health and quality of life among patients with CKD.

The diagnosis of CKD followed the medical records of each participant, according to nephrological evaluation and clinical classification of ESRD prior to transplantation. Oral conditions were diagnosed based on clinical criteria recommended by the SB2000 guidelines for DMFT and CPI assessments. Quantitative variables, such as age, DMFT, CPI, and OHIP‐14 scores, were primarily identified as continuous.

### 2.10. Bias

To reduce selection bias, all patients with CKD awaiting kidney transplantation at the study center during the data collection period were invited to participate, minimizing the risk of including a nonrepresentative sample. Standardized instruments were used for data collection, including oral clinical examinations performed by calibrated examiners and the OHIP‐14 questionnaire, which reduces information bias. Examiner training and the use of objective diagnostic criteria (DMFT index, Community Periodontal Index) ensured consistency and reliability in clinical assessments. Data were analyzed using appropriate statistical methods to control for confounding factors, including sociodemographic variables and lifestyle habits, helping to reduce confounding bias.

### 2.11. Statistical Analysis

The SPSS 20.0 software was used for statistical analysis, adopting a level of significance of 5%. Since the total OHIP‐14 score did not show a normal distribution, the Mann–Whitney test for comparison of two groups and the Kruskal–Wallis test for comparison of two or more groups in independent samples were used to evaluate the association between data.

Subgroups were analyzed according to sociodemographic and clinical characteristics, using the nonparametric Mann–Whitney and Kruskal–Wallis tests as appropriate, considering the nonnormal distribution of OHIP‐14 scores. Formal interactions between variables were not assessed due to the cross‐sectional design of the study. Missing data were handled using a complete‐case analysis without imputation, as the proportion of missing values was low. Given the use of a nonprobabilistic convenience sample, no sampling adjustments were applied, and the results should be interpreted with caution regarding generalizability. Formal sensitivity analyses were not performed, but additional comparisons between clinical subgroups were conducted to assess the consistency of the findings.

## 3. Results

The sample of this study consisted of 80 patients with end‐stage CKD who would undergo kidney transplantation. Most patients were female (mean age of 43.21 ± 11.68 years), married and white and had a maximum household income between one and three minimum wages and complete secondary education, with most of them having up to nine complete years of schooling (Table 1).

**Table 1:** Socioeconomic‐demographic profile of the participants in the study.

Variable		*N* (%)
Sex	Female	43 (53.8)
	Male	37 (46.2)
Marital status	Married/stable union	57 (71.3)
	Single	16 (20)
	Divorced	06 (7.5)
	Widowed	01 (1.2)
Race	White	31 (38.8)
	Brown	28 (35)
	Black	21 (26.2)
Household income (dichotomized)	1 to 3 MW	51 (63.8)
	>3 MW	29 (36.2)
Educational level	Illiterate	3 (3.8)
	Incomplete elementary school	9 (11.2)
	Complete elementary school	5 (6.3)
	Incomplete high school	10 (12.5)
	Complete high school	28 (35)
	Incomplete higher education	10 (12.4)
	Complete higher education	13 (16.3)
	Postgraduation	2 (2.5)
Educational level (dichotomized)	≤9 years of schooling (complete high school)	55 (68.8)
	>9 years of schooling	25 (31.2)

Total	—	80 (100)

Regarding harmful habits, 25 patients (31.2%) had experience with cigarettes (smokers and former smokers) and 50 (62.5%) with alcoholic beverages (drinkers and former drinkers) (Table 2).

**Table 2:** Distribution of harmful habits among the participants in the study.

Variable		*N* (%)
Smoking	Smoker	4 (5)
	Former smoker	21(26.2)
	Never smoked	55 (68.8)
Alcohol consumption	Drinker	2 (2.5)
	Former drinker	48 (60.0)
	Never drank	30 (37.5)

Total	—	80 (100)

*Note*: Source: Data of the study.

With respect to medical history, most patients were on RRT, including hemodialysis (71/74; 95.9%) and peritoneal dialysis (3/74; 4.1%). The average duration of RRT was 38.36 ± 36.926 months (range: 1 month to 180 months). The majority of patients received transplants from living donors, and hypertension was the main underlying cause of kidney failure (Table 3). Among the comorbidities observed, all patients had at least one disease, with hypertension, DM, and anemia being the most prevalent (Table 4).

**Table 3:** Distribution of medical data among the participants in the study.

Variable		*N* (%)
Renal replacement therapy	No	6 (7.5)
	Yes	74 (92.5)
Type of transplant	Living	45 (56.2)
	Deceased	35 (43.8)
Underlying cause of kidney failure	Hypertension	23 (28.8)
	Diabetes mellitus	11 (13.8)
	Undefined	11 (13.8)
	Polycystic kidneys	6 (7.5)
	Focal segmental glomerulosclerosis	6 (7.5)
	Glomerulonephritis	5 (6.3)
	IgA nephropathy	4 (5.0)
	Kidney stones	3 (3.7)
	Lupus erythematosus	2 (2.5)
	Alport syndrome	2 (2.5)
	One kidney	2 (2.5)
	Ureteral stricture	2 (2.5)
	Use of anti‐inflammatory	1 (1.2)
	Hydronephrosis	1 (1.2)
	Urinary infection	1 (1.2)

Total	—	80 (100)

*Note*: Source: Data of the study.

**Table 4:** Distribution of comorbidities among the participants in the study.

Variable		*N* (%)
Comorbidities	No	11 (13.7)
	Yes	69 (86.3)
Hypertension	No	21 (26.2)
	Yes	59 (73.8)
Diabetes mellitus	No	65 (81.3)
	Yes	15 (18.7)
Anemia	No	45 (56.3)
	Yes	35 (43.7)
Depression	No	78 (97.5)
	Yes	2 (2.5)
Anxiety	No	69 (86.3)
	Yes	11 (13.7)
Systemic lupus erythematosus	No	79 (98.8)
	Yes	1 (1.2)
Gastritis	No	79 (98.8)
	Yes	1 (1.2)
History of stroke	No	79 (98.8)
	Yes	1 (1.2)
History of infarction	No	78 (97.5)
	Yes	2 (2.5)

Total	—	80 (100)

*Note*: Source: Data of the study.

The patients were asked whether they had received information about the need for dental treatment before transplantation, and most of them reported that they did not receive this information at any time during the pretransplant preparation. The patients were also asked whether they had undergone dental treatment as part of the multidisciplinary preparation for kidney transplantation. Twenty patients (25%) had undergone dental treatment, with restorative and periodontal treatments being the most commonly performed (Table 5).

**Table 5:** Dental treatment for transplant preparation.

Variable		*N* (%)
Received information about the need for dental treatment before transplantation	No	55 (68.8)
	Yes	25 (31.2)
Underwent dental treatment as part of multidisciplinary preparation for kidney transplantation	No	60 (75)
	Yes	20 (25)
Restorative treatment	No	8 (40)
	Yes	12 (60)
Periodontal (gingival) treatment	No	5 (25)
	Yes	15 (75)
Surgery (tooth extraction)	No	15 (75)
	Yes	5 (25)
Endodontic treatment	No	18 (90)
	Yes	2 (10)

Total	—	80 (100)

*Note*: Source: Data of the study.

Regarding the oral health parameters evaluated before kidney transplantation, the majority of the sample had a high DMFT index and altered CPI. Caries experience (DMFT): Mean DMFT in the sample was 15.33 ± 9.37. Distribution: very low < 5:11/80 (13.8%); low 5–8.8:7/80 (8.7%); moderate 9–13.9:15/80 (18.7%); and high > 13.9:47/80 (58.8%). Pretransplant periodontal status (CPI) for context: altered: 31/73 (42.5%); calculus: 24/80 (30.0%) (Table 6).

**Table 6:** Oral health condition of the patients before kidney transplantation.

Variable		*N* (%)
DMFT index (dichotomized)	Very low (<5)	11 (13.8)
	Low (5–8.8)	7 (8.7)
	Moderate (9–13.9)	15 (18.7)
	High (>13.9)	47 (58.8)
Pretransplant CPI	(0) Healthy sextant	42 (52.5)
	(1) Sextant with bleeding	1 (1.3)
	(2) Calculus	24 (30)
	(3) Pocket depth 4 to 5 mm	5 (6.2)
	(4) Pocket depth ≥6 mm	1 (1.3)
	Sextant excluded	7 (8.7)
Pretransplant CPI (dichotomized)	Healthy	42 (57.5)
	Altered	31 (42.5)

Total	—	80 (100)

*Note*: Source: Data of the study.

Analysis of the impact of oral health on the patient’s quality of life showed that the main changes in the OHIP‐14 were related to embarrassment due to oral problems (question 10), discomfort while eating (question 4), and the presence of pain (question 3) (Table 7).

**Table 7:** Impact of oral health on the patient’s quality of life (OHIP‐14) during the pretransplantation phase based on participant responses.

Question	0 *N* (%)	1 *N* (%)	2 *N* (%)	3 *N* (%)	4 *N* (%)
1. Have you had difficulty pronouncing any words because of problems with your mouth or teeth?					
	73 (91.3)	1 (1.3)	5 (6.1)	1 (1.3)	0 (0)
2. Have you felt that your sense of taste has worsened because of problems with your mouth or teeth?					
	68 (85.0)	5 (6.2)	5 (6.2)	1 (1.3)	1 (1.3)
3. Have you had pain in your mouth or teeth?					
	61 (76.3)	10 (12.5)	9 (11.2)	0 (0)	0 (0)
4. Have you found it uncomfortable to eat any food because of problems with your mouth or teeth?					
	60 (75.0)	11 (13.8)	9 (11.2)	0 (0)	0 (0)
5. Are you self‐conscious about your mouth or teeth?					
	68 (85.0)	6 (7.4)	5 (6.3)	0 (0)	1 (1.3)
6. Do you feel tense because of problems with your mouth or teeth?					
	69 (86.2)	6 (7.5)	4 (5.0)	0 (0)	1 (1.3)
7. Has your diet been unsatisfactory because of problems with your mouth or teeth?					
	71 (88.7)	8 (10.0)	1 (1.3)	0 (0)	0 (0)
8. Have you had to interrupt meals because of problems with your mouth or teeth?					
	71 (88.7)	8 (10.0)	1 (1.3)	0 (0)	0 (0)
9. Have you found it difficult to relax because of problems with your mouth or teeth?					
	72 (90)	5 (6.3)	3 (3.7)	0 (0)	0 (0)
10. Are you a bit embarrassed because of problems with your mouth or teeth?					
	57 (71.2)	5 (6.3)	11 (13.8)	2 (2.4)	5 (6.3)
11. Do you get a bit irritated with other people because of problems with your mouth or teeth?					
	80 (100)	0 (0)	0 (0)	0 (0)	0 (0)
12. Have you had difficulty doing your usual tasks because of problems with your mouth or teeth?					
	76 (95.0)	2 (2.5)	2 (2.5)	0 (0)	0 (0)
13. Have you felt that life in general was less satisfying because of problems with your mouth or teeth?					
	73 (91.3)	3 (3.7)	3 (3.7)	0 (0)	1 (1.3)
14. Have you been totally unable to function because of problems with your mouth or teeth?					
	79 (98.7)	1 (1.3)	0 (0)	0 (0)	0 (0)

*Note*: Source: Data of the study.

Table 8 presents the analysis of the association between sociodemographic and clinical variables and Oral Health‐Related Quality of Life (OHRQoL), measured by the total OHIP‐14 score at the pre‐intervention time point. Among the sociodemographic determinants, educational level showed a statistically significant association with quality of life (*p*  = 0.020). Patients with lower education (≤9 years of schooling) had a higher median (median = 2.00) compared to those with a higher level of education, whose median was zero (0.00). Regarding systemic clinical variables, the presence of RRT significantly influenced the impact on oral health (*p*  = 0.030), with patients on dialysis reporting higher scores (median = 1.00) than those undergoing conservative treatment. Other comorbidities, including diabetes and hypertension, as well as the presence of anxiety or depression disorders, did not show a significant correlation with OHIP‐14 scores in this sample. Regarding dental variables, objective clinical indices, such as CPI (periodontal condition) and DMFT (decay history), did not show a statistical association with the perception of quality of life (*p*  > 0.05).

**Table 8:** Comparison between variables and OHIP‐14 (pretransplant).

Variable		*N* (%)	Median	Interquartile range	*p*
Sex	Female	43	1.00	5.00	0.685^c^
	Male	37	1.00	3.00	—
Household income (dichotomized)	1 to 3 MW	51	1.00	5.00	0.319^c^
	>3 MW	29	1.00	3.00	—
Educational level (dichotomized)	≤9 years of schooling (complete high school)	55	2.00	5.00	0.020^c a^
	>9 years of schooling	25	0.00	2.00	—
Present or past smoking	No	55	1.00	3.00	0.282^c^
	Yes	25	2.00	5.00	—
Present or past alcohol drinking	No	30	1.00	4.00	0.372^c^
	Yes	50	1.00	4.00	—
RRT	No	6	0.00	1.00	0.030^c a^
	Yes	74	1.00	4.00	—
Comorbidities	No	11	1.00	2.00	0.897^c^
	Yes	69	1.00	4.00	—
Hypertension	No	21	1.00	3.00	0.779^c^
	Yes	59	1.00	4.00	—
Diabetes mellitus	No	65	1.00	4.00	0.970^c^
	Yes	15	1.00	2.00	—
Anemia	No	45	1.00	3.00	0.960^c^
	Yes	35	1.50	4.00	—
Depression	No	78	1.00	3.00	0.411^c^
	Yes	2	3.50	—	—
Anxiety	No	69	1.00	3.00	0.087^c^
	Yes	11	2.00	5.00	—
Underwent dental treatment as part of multidisciplinary preparation for kidney transplantation	No	60	1.00	3.00	0.053^c^
	Yes	20	3.00	4.00	—
Received information about the need for dental treatment before transplantation	No	55	1.00	3.00	0.062^c^
	Yes	25	2.00	4.00	—
CPI (dichotomized)	Healthy	42	1.00	3.00	0.565^c^
	Altered	31	2.00	5.00	—
DMFT index (dichotomized)	Very low (<5)	11	2.00	3.00	0.579^b^
	Low (5–8.8)	7	1.00	2.00	—
	Moderate (9–13.9)	15	1.00	4.00	—
	High (>13.9)	47	2.00	5.00	—
Marital status	Single	16	1.00	5.00	0.657^b^
	Married/Stable union	57	1.00	3.00	—
	Divorced	6	0.00	—	—
	Widowed	1	6.00	—	—
Race (dichotomized)	White	31	1.00	2.00	0.054^c^
	Brown/Black	49	2.00	6.00	—
Total	—	80	—	—	—

*Note*: Source: Data of the study. Multiple comparisons tests.

Abbreviations: MW, minimum wage; RRT, renal replacement therapy.

^a^Valor de p para os testes (Teste de Mann–Whitney e o Kruskal–Wallis para as comparações múltiplas). ^a^Statistically significant.

^b^Kruskal–Wallis test.

^c^Mann–Whitney test.

Table 9 presents a comparison between the seven domains that comprise the OHIP‐14 instrument. Analysis using the Friedman test revealed a statistically significant difference between the evaluated dimensions (*p*  < 0.001), indicating that the impact of oral health on quality of life does not occur uniformly across different aspects of patients’ lives. Although the median was zero (0.00) for all domains individually, the dispersion analysis (interquartile range) allows for the identification of the areas of greatest impairment. The psychological disability domain showed the greatest variability (interquartile range = 1.75), suggesting it is the main component contributing to the total score, followed by the physical pain domain (interquartile range = 1.00). In contrast, the dimensions of functional limitation, psychological discomfort, physical disability, social disability, and social disadvantage presented a median and interquartile range of zero, indicating a negligible impact of these aspects on the perception of quality of life in this specific sample. The total OHIP‐14 score resulted in a median of 1.00, confirming a generally positive perception of oral health‐related quality of life, but with specific impairments concentrated in the psychological and pain spheres.

**Table 9:** Analysis of the OHIP‐14 domains.

Domains evaluated^b^	Questions	Median of the sum	Interquartile range of the sum	*p*‐Value^a^
Functional limitation	1, 2	0.00	0,00	<0.001
Physical pain	3, 4	0,00	1,00	—
Psychological discomfort	5, 6	0,00	0,00	—
Physical disability	7, 8	0,00	0,00	—
Psychological incapacity	9, 10	0,00	1,75	—
Social disability	11, 12	0,00	0,00	—
Social disadvantage	13, 14	0,00	0,00	—
OHIP‐14	—	1,00	5,00	—

^a^ *p* ‐value of the Friedman test.

^b^Schmalz et al.[11].

## 4. Discussion

Analysis of the socioeconomic‐demographic profile of the present sample showed that most patients were women, adults, married, and white, who had a maximum income of three minimum wages, completed high school, and had a mean age of 43.21 ± 11.68 years. The findings agree with previous studies in terms of sex [16, 17], race [16], household income [3, 18], marital status [19, 20], and age [3, 16–18, 21]. On the other hand, there was disagreement with some studies in terms of sex [3, 18, 21], race [3, 18], and educational level [16, 18]. From an interpretative standpoint, this demographic profile suggests that socioeconomic constraints and intermediate educational levels may contribute to poorer oral health indicators and greater vulnerability to chronic disease progression.

Studies have demonstrated an increased risk of CKD among smokers and former smokers [16], who are more likely to develop ESRD, a fact that renders this impact more important when the duration of the habit is measured [22]. The present study corroborated previous findings regarding a history or presence of smoking by demonstrating experience with cigarettes in a considerable part of the sample. Given the established inflammatory burden associated with smoking, this behavioral profile may partially explain the high prevalence of comorbidities and oral health impairment found in our participants. Smoking may therefore act as a confounding factor, reinforcing the importance of careful adjustment in future analyses.

Controversy exists regarding the relationship between a history of alcohol consumption, which was reported by most patients of the present sample, and CKD. One study found that alcohol consumption reduced the risk of developing CKD [16], while another observed that this consumption was associated with a higher risk of CKD, which increased almost twice after adjusting for age, sex, use of nonsteroidal inflammatory drugs, and baseline comorbidities [23]. This contradiction may be due to the lack of reliability in assessing this habit since data on alcohol consumption were collected by self‐report in most studies, reducing the accuracy of these data because of the influence of memory bias and type of questionnaire, among other factors. In our sample, the high frequency of alcohol history should therefore be interpreted with caution, as misclassification bias may have influenced the associations with oral health outcomes.

Regarding therapeutic modality, most participants in the present study were on RRT, with hemodialysis being the main type, in agreement with previous studies [20, 24–26]. Furthermore, the duration of RRT was also similar to the present study, with a mean duration of up to 24 months [26], 60 months [20], 33 months [25], and 36 months [19]. The predominance of hemodialysis and the relatively long treatment duration may have contributed to the negative impact on quality of life identified in OHIP‐14 domains. Hemodialysis regimens impose dietary restrictions, metabolic fluctuations, and fatigue, all of which may exacerbate oral symptoms and reduce daily functioning.

In the present study, most transplanted organs came from living donors. This finding can be explained by the fact that living donors provide better quality organs when compared to deceased donors [27].

With respect to the underlying cause of ESRD, hypertension was the main disease reported, corroborating previous studies that indicate hypertension as the main comorbidity associated with CKD [21, 24, 28, 29]. The second most prevalent condition was DM, in agreement with the literature that reported these two conditions to be the main factors associated with the progression of CKD and subsequent ESRD [5, 16, 18, 24, 30]. On the other hand, partially contradicting the findings of the present study, other authors identified glomerulonephritis and DM as the main underlying causes of CKD, while hypertension was the third most cited disease [21]. In the context of our sample, the predominance of hypertension and DM has important implications, as these comorbidities are known to intensify periodontal inflammation and caries activity, possibly contributing to higher DMFT and CPI values.

It should be noted that chronic inflammatory processes are common in patients with CKD and ESRD and are generally accompanied by comorbidities such as hypertension and DM, which represent a complicating factor for the patient. These complications may be related to inflammatory markers that increase the wear and tear on kidney function. A study evaluating the correlation between serum levels of inflammatory markers (C‐reactive protein [CRP], TNF‐α, and IL‐1b) and comorbidities in patients with ESRD found significantly higher serum concentrations of CRP and TNF‐α in patients with hypertension or diabetes or with both comorbidities when compared to patients without these conditions, while serum IL‐1b concentrations did not differ significantly [21]. These results support the hypothesis of the involvement of inflammatory processes as complicating factors of the renal condition in patients with ESRD. This biological pathway may also partially explain the association between systemic conditions and the compromised oral health observed in the present study, reinforcing the bidirectional relationship between systemic inflammation and oral disease.

In the present study, most patients did not receive information about the need for dental treatment before transplantation, nor had they undergone any dental treatment as part of pretransplant preparation. This finding is consistent with the fact that there is a lack of knowledge about the importance of oral health among professionals. Within this context, one study demonstrated a lack of knowledge regarding the oral health of patients with ESRD among the nursing team and poor attitudes that prevented patients from receiving appropriate information [31]. It is worth noting that crown‐root scaling has been reported to be strongly associated with a lower risk of progression to ESRD and is therefore an important prophylactic strategy to improve the prognosis of CKD [32]. These facts highlight the importance of including the dentist in the multidisciplinary kidney transplant team. Clinically, this reinforces the urgent need for structured oral health protocols in transplant centers and underscores that the absence of pretransplant dental care may be contributing to the poorer OHIP‐14 scores observed.

Regarding the oral health‐related parameters of patients prior to transplantation, most patients of the present sample had a high DMFT index, and a significant percentage had an altered CPI. These findings corroborate previous studies that demonstrated high DMFT and CPI indices in patients with CKD and ESRD; however, there were small variations in these indices across studies [31, 33–35]. These results demonstrate that oral disease burden remains substantial in CKD populations even in pretransplant settings, reinforcing the consistency of our findings with the broader literature.

Previous studies showed a high prevalence of caries among patients with ESRD, with significantly higher DMFT indices in patients with low education and less time on dialysis [31]. Furthermore, a systematic review reported a high DMFT index in hemodialysis patients with DM. Regarding the CPI, some authors found a significantly higher index in patients with CKD and DM. In contrast, one study cited in the review reported that this significance was borderline between diabetic and nondiabetic patients with CKD, while another study did not find a significant association between CPI and DM in CKD patients [36].

Quality of life is related to a series of factors, with oral health status being particularly important. Thus, oral health‐related quality of life is an integral part of general health and can have a great impact on the individual’s condition, including functional and emotional well‐being [11, 13, 14]. Furthermore, oral health‐related quality of life is considered a multidimensional model that provides information about the influence of factors such as oral function, orofacial pain, orofacial appearance, and psychosocial impact and is of great clinical relevance for patients undergoing RRT [11].

The oral health condition is known to potentially influence the quality of life of patients undergoing RRT. It is therefore recommended that dental criteria be assessed for interdisciplinary treatment by medical and dental teams in order to reduce the risk of infections, systemic complications, and malnutrition, thus improving the individual social, emotional, and physical functioning of a patient undergoing RRT. However, it is noteworthy that the true impact of oral health on the mortality of these patients is controversial. Within this context, few studies have confirmed the impact of periodontal indices on the quality of life of patients with ESRD and a history of tooth loss as an influential factor. Thus, greater control of oral health is necessary, as well as a better understanding of the need for multidisciplinary dental treatment [11].

Within this context, the impact of oral health status on the quality of life of individuals can be assessed using instruments such as the OHIP‐14 [13, 14]. In the present study, the main changes observed in the OHIP‐14 involved physical (pain and discomfort) and psychological aspects. These findings are in line with previous studies showing that CKD and subsequent ESRD have a major impact on different domains of oral health, consequently affecting daily functions in terms of physical, social, and psychological aspects related to oral health [37].

Another study applying the OHIP‐14 identified as the most prevalent domains psychological impacts represented by the items “being self‐conscious, having difficulty to relax, being ashamed, and having a less satisfactory life,” as well as pain and discomfort represented by “painful symptoms in the mouth and feeling uncomfortable to eat any foods” [13]. The results of the present study corroborate these findings regarding the psychological impact represented by the item “being ashamed” and pain and discomfort represented by “pain in the mouth and feeling uncomfortable to eat any foods.”

Comorbidities in general did not seem to influence OHIP‐14 scores; however, depression and anxiety alone had a negative impact on the quality of life of the participants, indicating an impact on the psychological and/or psychosocial domain. Previous authors reinforced this psychosocial aspect by demonstrating a negative correlation with quality of life assessed by the OHIP‐14 [38]. Likewise, another study applying the OHIP‐14 showed that mild, moderate, and severe periodontitis were significantly associated with worsening quality of life, with an impact on the psychological disability domain. Severe periodontitis also significantly impacted the pain and physical and psychological disability domains [39].

Regarding the variables represented in this study and their influence on quality of life related to oral health, it was observed that these varied between a negative impact and influence in front of the OHIP‐14, in addition to statistical significance when related to the questionnaire. However, in contrast to our results, one study did not find a significant correlation of the duration of RRT (hemodialysis) or DMFT and CPI indices with the OHIP‐14. On the other hand, the need for dental treatment and self‐assessment of poor oral health status were significantly associated with the OHIP‐14. Furthermore, there was a relationship between the demographic variables sex and years of schooling and the OHIP‐14 [13]. Some authors also found a significant correlation of the CPI with the total OHIP‐14 score, as well as with the individual domains of functional limitation, physical pain, physical disability, and deficiency. However, psychosocial factors related to unsatisfactory diet, irritation, and embarrassment were not significantly correlated with the CPI [14].

In contrast to the present study, one study found a negative impact on oral health‐related quality of life in younger patients when compared to older patients [39]. Another study reported a significant correlation between the DMFT index and the OHIP‐14, in which the higher the DMFT index, the greater the negative impact on the patients’ quality of life. All OHIP‐14 domains showed a positive correlation with the DMFT index, except for psychological parameters [37].

Studies reiterate that CKD directly affects the oral health of individuals since they tend to have more decayed teeth and significant periodontal alterations [4, 8], which can have a negative impact on the quality of life of these patients [2, 4, 9, 10]. Within this context, the results of the present study highlight the importance of the dentist in the multidisciplinary transplant team so that oral health strategies can be designed for better controlling and/or reducing the risk of organ transplant failure.

Several potential confounding factors may have influenced the associations observed in this study. Variables such as age, CKD severity and duration, time on RRT, and comorbidities like diabetes and hypertension—conditions known to exacerbate periodontal disease and caries—may partially explain the elevated DMFT and CPI values. Behavioral and socioeconomic aspects (smoking, alcohol intake, oral hygiene, education, and income), as well as inflammatory status, polypharmacy, and pretransplant immunosuppression, may also affect oral conditions and OHIP‐14 scores. Since analytical adjustments for these variables were not performed, residual confounding remains a relevant limitation and should be addressed in future multivariable studies.

Another limitation is the use of the global DMFT index without separating its components (decayed, missing, and filled), which restricts a more detailed understanding of oral health profiles. As each component has distinct clinical and epidemiological implications, future research should analyze them individually to better guide treatment planning and public health strategies for CKD patients.

Despite its contributions, this study is subject to sources of bias. Convenience sampling may have introduced selection bias, and self‐reported habits such as smoking and alcohol intake are prone to recall bias. The cross‐sectional design precludes causal inference, and the absence of power calculation may reduce estimate precision. Nevertheless, the findings align with previous evidence showing high oral disease burden and its impact on quality of life in CKD populations. Results are mostly generalizable to adults undergoing RRT in comparable settings, although broader extrapolation should be done with caution. Longitudinal studies with probabilistic sampling and analytical models adjusted for systemic, behavioral, and inflammatory variables are needed to better elucidate causal pathways and support targeted preventive strategies.

## 5. Conclusion

The findings of this study reveal a scenario of serious diagnostic and therapeutic neglect in the oral health of patients in the prerenal transplant phase. The coexistence of high caries indices (DMFT 15.33) and periodontal disease (42.5% altered CPI) with a predominantly positive perception of quality of life indicates that patients tend to underestimate oral risks in the face of the severity of systemic disease. Clinically, this represents a high risk of infectious foci that can compromise the success of the transplant and the biological stability of the individual. Statistical analysis demonstrated that the impact on quality of life does not occur uniformly, being determined by specific socioeconomic and clinical factors, such as low educational level (*p*  = 0.020) and dependence on RRT (*p*  = 0.030). Specifically, the dimensions of physical pain and psychological disability (such as shame) were the areas that most drained patients’ well‐being, as evidenced by the greater variability and scores in the domains of mouth pain, discomfort when eating, and embarrassment due to oral problems.

Given the high rate of misinformation (68.8%) and low adherence to dental treatment (25%) before transplantation, it is imperative that health centers establish mandatory and early dental screening protocols. The effective integration of the dentist into the nephrology team is essential to mitigate systemic infectious risks and, fundamentally, to ensure the necessary psychosocial support for those most vulnerable during the transplantation process.

## Author Contributions

**Sérgio Henrique Gonçalves de Carvalho**: conceptualization, data curation, research, project management, manuscript writing original. **Jackeline Mayara Inácio Magalhães**: methodology, data presentation design, writing – review and editing. **Gustavo Gomes Agripino**: formal analysis, software development, implementation and testing, supervision. **Dmitry José de Santana Sarmento**: data curation, methodology, supervision, data and experiment validation. **Marina Gallottini**: data curation, supervision. **Gustavo Pina Godoy**: conceptualization, methodology, supervision, data and experiment validation, writing – review and editing.

## Funding

The authors state that this study did not receive funding from any institution or funding agency, having been entirely funded by the authors themselves.

## Conflicts of Interest

The authors declare no conflicts of interest.

## Data Availability

The data that support the findings of this study are available from the corresponding author upon reasonable request.
